# A Low-Level Perceptual Correlate of Behavioral and Clinical Deficits in ADHD

**DOI:** 10.1162/cpsy_a_00018

**Published:** 2018-10

**Authors:** Andra Mihali, Allison G. Young, Lenard A. Adler, Michael M. Halassa, Wei Ji Ma

**Affiliations:** 1 Center for Neural Science, New York University, New York, New York, USA; 2 Department of Psychology, New York University, New York, New York, USA; Department of Psychiatry, NYU School of Medicine, New York, New York, USA; Department of Psychiatry, NYU School of Medicine, New York, New York, USA; Department of Brain and Cognitive Science, MIT, Boston, Massachusetts, USA; 6 Center for Neural Science, New York University, New York, New York, USA; 7 Department of Psychology, New York University, New York, New York, USA

**Keywords:** ADHD, visual perception, variability, psychophysics, executive function, task-switching

## Abstract

In many studies of attention-deficit hyperactivity disorder (ADHD), stimulus encoding and processing (perceptual function) and response selection (executive function) have been intertwined. To dissociate deficits in these functions, we introduced a task that parametrically varied low-level stimulus features (orientation and color) for fine-grained analysis of perceptual function. It also required participants to switch their attention between feature dimensions on a trial-by-trial basis, thus taxing executive processes. Furthermore, we used a response paradigm that captured task-irrelevant motor output (TIMO), reflecting failures to use the correct stimulus-response rule. ADHD participants had substantially higher perceptual variability than controls, especially for orientation, as well as higher TIMO. In both ADHD and controls, TIMO was strongly affected by the switch manipulation. Across participants, the perceptual variability parameter was correlated with TIMO, suggesting that perceptual deficits are associated with executive function deficits. Based on perceptual variability alone, we were able to classify participants into ADHD and controls with a mean accuracy of about 77%. Participants’ self-reported General Executive Composite score correlated not only with TIMO but also with the perceptual variability parameter. Our results highlight the role of perceptual deficits in ADHD and the usefulness of computational modeling of behavior in dissociating perceptual from executive processes.

## INTRODUCTION

In ADHD, the behavioral deficits captured by self-reports and collateral reports have been attributed to differences in attention, executive function, and lower level processes, including perceptual function. In the realm of visual attention, differences in accuracy or reaction time (RT) have been found in some visual search tasks but not in others (for a review, see Mullane & Klein, 2008). No consistent deficits have been found when probing selective attention with visuo spatial orienting tasks (Cubillo et al., 2010; C. L. Huang-Pollock & Nigg, 2003; Roberts, Ashinoff, Castellanos, & Carrasco, 2017; Rubia et al., 2010). ADHD patients tend to have worse executive function than controls (Boonstra, Oosterlaan, Sergeant, & Buitelaar, 2005; Castellanos & Tannock, 2002; Kofler et al., 2013; Willcutt, Doyle, Nigg, Faraone, & Pennington, 2005) predominantly in response execution and inhibition (Barkley, 1997; Booth et al., 2005; Casey et al., 1997) but also in working memory and switching between stimulus-response rules (Cepeda, Cepeda, & Kramer, 2000; Halleland, Haavik, & Lundervold, 2012; Homack, 2004; King, Colla, Brass, Heuser, & von Cramon, 2007).

While some researchers believe executive function impairments to be primary in ADHD, others acknowledge that they are neither necessary nor sufficient to cause the disorder (Boonstra et al., 2005; Willcutt et al., 2005). More specifically, yet others suggest that ADHD impairments are a combination of deficits in high-level and “low-level processes” (Castellanos et al., 2008; Gonen-Yaacovi et al., 2016; Killeen, Russell, & Sergeant, 2013; Rommelse et al., 2007; Sergeant, Geurts, & Oosterlaan, 2002; Sonuga-Barke & Castellanos, 2007). These low-level processes entail arousal (Sergeant, 2005), and relatedly, accumulation of evidence (Karalunas & Huang-Pollock, 2013), timing (Nigg & Casey, 2005), or reward sensitivity (see I. Ma, van Duijvenvoorde, & Scheres, 2016; Sonuga-Barke, 2003, for reviews). It should be kept in mind that ADHD might be a heterogenous disorder (Fair, Bathula, Nikolas, & Nigg, 2012; Nigg, Willcutt, Doyle, & Sonuga-Barke, 2005), and different causes might apply to different deficits.

Here, we extend the examination of low-level processes to perceptual encoding. Behavioral studies that examined the quality of perceptual encoding in ADHD in the absence of attentional or executive involvement have found small and inconsistent differences (see Fuermaier et al., 2017, for a review). On the other hand, other investigations have found evidence for self-reported impairments in perceptual function in ADHD participants (Bijlenga, Tjon-Ka-Jie, Schuijers, & Kooij, 2017; Micoulaud-Franchi et al., 2015) or in the general population with ADHD traits (Panagiotidi, Overton, & Stafford, 2018), as well as deficits in color processing and self-reported visual function in ADHD (Kim, Chen, & Tannock, 2014). These findings are not necessarily contradictory, as perceptual deficits might emerge when attention or executive function is simultaneously taxed.

Therefore, we believe it is important to use a task that taxes both perceptual function and either attention and/or executive function but that allows for a dissociation of the respective processes. This dissociation is difficult, as has been described in the study of autism (Robertson & Baron-Cohen, 2017). In ADHD, there have been a few attempts to dissociate perceptual function from attention within a single task (Kim, Al-Haj, Fuller et al., 2014; McAvinue et al., 2012; Stevens et al., 2012). For example, Stevens et al. (2012) compared performance on digit reports with or without distractors (letters surrounding the digits) and found that ADHD participants had lower performance only when distractors were present. However, spatial covert attention was similar across ADHD and controls, leading the authors to suggest that perceptual interference or crowding is increased in ADHD.

It is still unknown whether perceptual function is impaired when executive function is simultaneously taxed. A study by Friedman-Hill et al. (2010) used a face discrimination task where they probed perceptual noise by manipulating distractor saliency and probed top-down executive control by parametrically manipulating discrimination difficulty. In difficult discriminations, the reaction time difference between high-salience and low-salience distractors was comparable in the children with ADHD to that in the healthy children and adults; however, in easy discriminations, children with ADHD were slower to respond when presented with low-salience distractors. These results suggest similar perceptual interference due to distractor salience in ADHD and controls but a higher threshold in ADHD for activating executive control of attention. A complication in the design of Friedman-Hill et al. (2010) is that face stimuli are high-dimensional and have content at many levels, complicating the separation between perceptual, attentional, and executive function. Another complication is that if the observer uses only two response keys in a task-switching paradigm, an error could be either due to a failure to switch or to a successful switch followed by a perceptual or attentional error (Ravizza & Carter, 2008).

Here, we attempted to characterize performance in early processes of perceptual encoding in ADHD and dissociate them from later response selection (executive processes) using a visuo-motor decision-making paradigm with task-switching, which avoids the complications listed above. By using a total of eight possible buttons out of which only two were relevant on a given trial, our response paradigm allowed for task-irrelevant motor output (TIMO), a new measure of executive control deficits. We defined a perceptual error as a press of the wrong button among the two relevant ones. We optimized the quantitative characterization of perceptual function by (a) using simple stimuli with feature dimensions orientation and color, thus minimizing high-level cognitive effects; (b) varying stimuli parametrically along a continuum to estimate psychometric curve parameters (standard in perceptual psychophysics but still relatively rare in the study of ADHD, Chen & Niemeier, 2017; Friedman-Hill et al., 2010; Kim, Al-Haj, Chen et al., 2014; Kim, Al-Haj, Fuller et al., 2014; Roberts et al., 2017; Stevens et al., 2012); and (c) using an efficient stimulus selection method to minimize the number of trials needed for accurate estimation of parameters (Acerbi, 2016). Broadly, our work follows a recent proposal to apply four levels of analysis to computational psychiatry: development of behavioral tasks, fitting of computational models, estimating parameters, and classification for diagnosis (Wiecki, Poland, & Frank, 2015).

## METHODS

### Approach

Twenty ADHD and 20 control adult participants took part in our experiment. Stimuli were two colored ellipses; each display contained one stimulus on the right of the fixation dot and one on the left. The participants performed yes–no discrimination of one of the ellipses (see Klein, 2001, for comparison with other psychophysical tasks). Specifically, the participants performed either fine orientation discrimination (was the cued ellipse clockwise or counterclockwise relative to vertical?) or fine color discrimination (was the cued ellipse more yellow or more blue relative to mid-level green?). The cue was 100% valid. In this task, participants had to rely on their internal memorized references, here for vertical and respectively the mid-level green in between the specific isoluminant yellow and blue values chosen.

**Figure 1.**
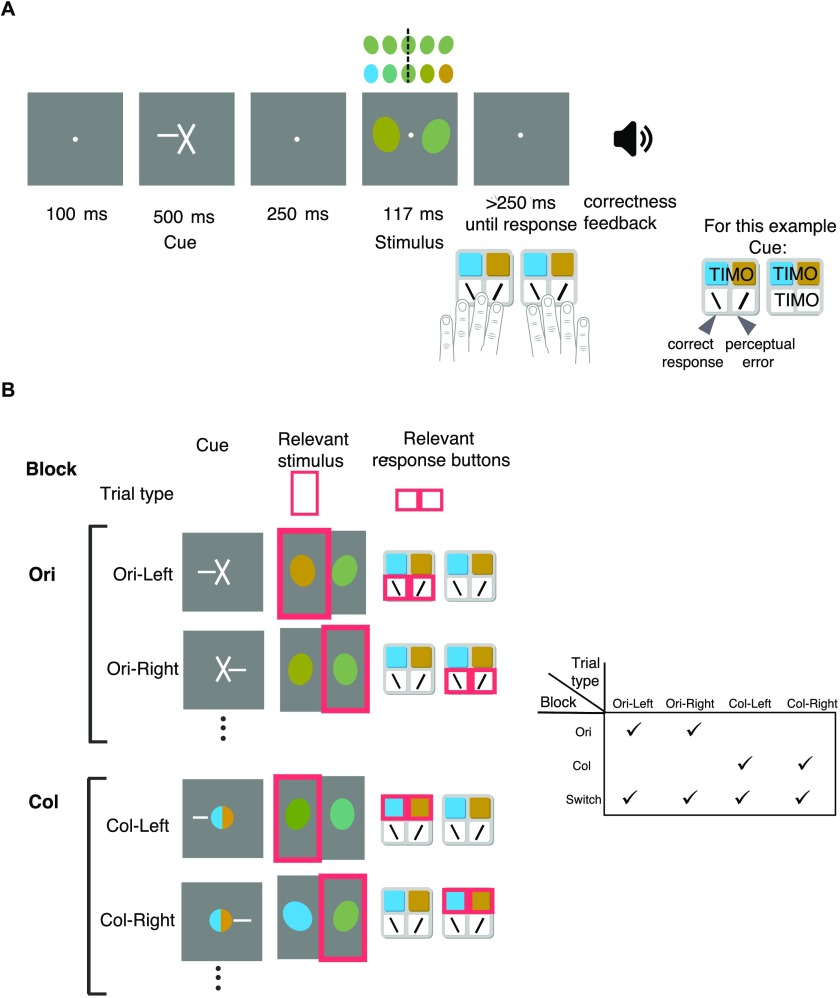
**Task design.** A) Trial sequence example. A feature dimension cue indicated whether orientation (cross)–depicted here–or color (colored circle) was relevant, while a simultaneous endogenous spatial cue (line segment) indicated which side (left or right) was relevant. Thus, the participant received one of four possible cue screens. We always chose the spatial cue randomly. The participant had to respond whether the orientation of the ellipse on the relevant side was clockwise or counterclockwise with respect to vertical or whether its color was more yellow or more blue, with the associated set of keys (left or right). The color and orientation continua are shown above the stimulus screen, with the dashed line at vertical and respectively mid-level green. To respond, the participant could press any one of eight keys, but only two were task-relevant on a given trial; the other six keys being considered task-irrelevant motor output. The participant received correctness feedback. B) Left: Cue–relevant stimulus–relevant response buttons pairings for the four types of trials as they arise from the four feature and spatial cue combinations (2 × 2). Relevant is marked with pink for visualization only. Pressing any other button would result in task-irrelevant motor output. Right: During Ori and Col blocks, only two types of trials are possible, while during Switch blocks, all four trial types are possible.

Every trial started with a symbolic feature dimension cue, informing the participant which feature dimension was relevant on that trial. Simultaneously presented was a spatial cue (a line segment), informing the participant which side of the screen was relevant on that trial (Figure 1A). To better detect failures of spatial or feature switching, we used a response paradigm in which, on each trial, only two of eight response keys were relevant, depending on the spatial and the feature cue; any other key press counted as TIMO. Separately in each condition and for each participant, we used a Bayesian adaptive method to select maximally informative stimuli (see “Target Stimulus Generation”). This method allowed us to estimate the psychometric curve parameters with relatively few trials.

Each participant experienced three types of blocks: Ori, Col, and Switch. In Ori blocks, the feature dimension cue was always orientation. The spatial cue was randomly chosen on each trial, yielding two possible trial types: Ori-Left and Ori-Right (Figure 1B). We analyzed the Ori-Left and Ori-Right trials together as the Ori condition. In Col blocks, the feature dimension cue was always color, and again the spatial cue was randomly chosen on each trial, yielding two possible trial types, Col-Left and Col-Right, which we grouped together for analysis into the Col condition. In Switch blocks, all four trial types were possible. We will refer to the orientation and color trials in switch blocks as the OriS and ColS conditions, respectively, and to the difference between no-switch and switch blocks as a difference in (executive) load.

**Figure 2.**
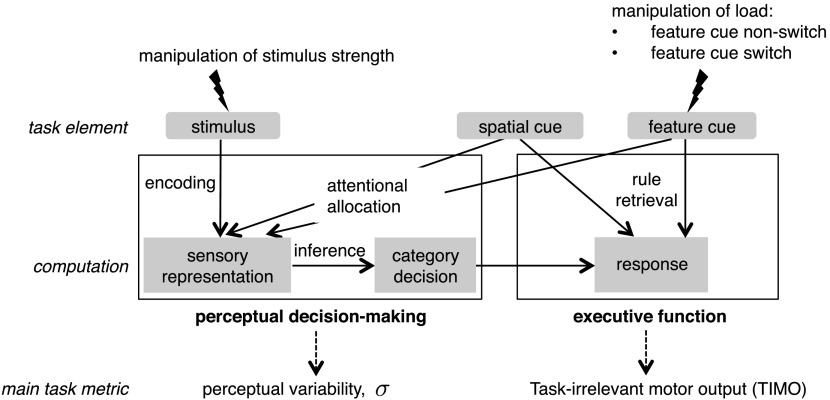
**Dissociation of perceptual and executive processes.** Schematic of the early perceptual encoding and late stimulus–response rule selection (executive) processes that may play a role in this task, and the corresponding task metrics.

An observer’s sequence of computations in the task can be conceptualized as a perceptual decision-making stage (stimulus encoding, affected by attention, and inference), followed by executive processing (rule retrieval and response execution, Figure 2). The parametric variation of stimulus strength allowed us to estimate perceptual variability *σ* (or noise, the inverse of slope/sensitivity) as a main metric of perceptual function, and the eight-button response paradigm allows us to estimate task-irrelevant motor output as a main metric of executive function. In addition, we characterized behavior using other psychometric curve parameters, median reaction time, and reaction time variability.

While usually a noise parameter (equivalent to our perceptual variability) in psychometric curves reflects a mix of sensory and decision noise (Gold & Ding, 2013), we believe that here the perceptual variability parameter for orientation and color is likely additionally modulated by attention. The Ori and Col conditions attempt to engage endogenous covert spatial attention, and the Switch conditions additionally engage attention to feature dimension. Previous studies showed modulation of psychometric curve parameters by attention, though either with larger stimulus eccentricies (usually equal or larger than 4 dva vs 2.5 dva here), in different tasks such as target detection (Bashinski & Bacharach, 1980), 2AFC orientation discrimination (Downing, 1988), color-change detection (Herman, Bogadhi, & Krauzlis, 2015), examined exogenous attention (Fuller & Carrasco, 2006), or with other stimulus strength manipulation, such as contrast (Ling & Carrasco, 2006; Pestilli, Viera, & Carrasco, 2007; for reviews, see Carrasco, 2011, 2014).

### Experiment

#### Participants

We recruited all participants through local advertisements, including flyers and newspaper and radio advertisements. Information on the participants is presented in Mihali, Young, Adler, Halassa, and Ma (2018, appendix). Participants in both groups were matched as much as possible by age, sex, and education (see Mihali et al., 2018, Appendix, Table A1). Twenty ADHD participants (12 female) of mean age 35.3 (*SD* 10.0, range 21 to 55) and 20 control participants (11 female) of mean age 32.5 (*SD* 6.1, range 19 to 44), with no statistical difference between their ages (Wilcoxon rank-sum test, *p* = 0.78), participated. Seventeen out of the 20 ADHD participants presented the combined subtype and three the inattentive subtype. All participants spoke English and had normal or corrected-to-normal vision. We asked every participant before they started if they were color-blind. One participant was excluded because of color-blindness. All participants provided informed consent. The study conformed to the Declaration of Helsinki and was approved by the Institutional Review Board of New York University School of Medicine.

#### Psychiatric Assessment and Diagnosis

None of the participants with ADHD were prescribed or took stimulant medication within 2 months of participating in the study. Participants with comorbid anxiety or unipolar depressive disorders were included as long as the symptoms at the time of evaluation were mild or in remission. Participants with bipolar disorders, psychotic disorders, substance use disorders, and neurologic disorders were excluded. For all adults, the diagnostic procedure included both clinician-administered and self-administered scales. A trained clinician assessed every participant using the Adult ADHD Clinician Diagnostic Scale (ACDS) v. 1.2, the Adult ADHD Investigator Symptom Rating Scale (AISRS), the Clinical Global Impressions–Severity of Illness (CGI-S) Scale, and the MINI International Neuropsychiatric Interview. All participants also completed the Adult ADHD Self-Report Scale (ASRS v. 1.1), the Adult ADHD Quality of Life (AAQoL) Scale, the World Health Organization Disability Assessment Schedule (WHODAS–II), and the Behavior Rating Inventory of Executive Function Adult Version (BRIEF-A). These scales have been extensively validated (L. Adler & Cohen, 2004; Kessler et al., 2005, 2006; Silverstein et al., 2018).

#### Apparatus

We displayed stimuli on a 23-inch (58.42 cm) Acer T232HL LCD monitor of resolution: 1920 × 1080 pixels and 60 Hz refresh rate (1 frame lasting 16.7 ms). We used a Kinesis Freestyle2 split keyboard. Participants used a head rest located at approximately 55 cm from the screen; this meant that 1 degree of visual angle (dva) subtended approximately 34 pixels. Stimulus presentation and response collection were controlled by a Windows computer running Matlab 7.1 (MathWorks, Massachusetts, USA) with Psychtoolbox3 (Brainard, 1997; Kleiner et al., 2007; Pelli, 1997) and EyeLink (Cornelissen, Peters, & Palmer, 2002).

For 10 out of 20 ADHD participants and 10 out of 20 control participants, we monitored their fixation and recorded their eye movements. The rationale for not eye tracking all participants was a mixture of lack of sufficient time on the participants’ side and balanced design on the experimenters’ side. The eye tracker was calibrated using the five-point calibration routine before every block. We recorded eye movements using a remote infrared video-oculographic system (EyeLink 1000 Plus; SR Research, Ltd, Mississauga, Ontario, Canada) with a 1 kHz sampling rate. We set the heuristic filtering option to “OFF.”

#### Stimuli

The background was mid-level gray (28.7 cd/m^2^). The stimuli were ellipses with area of 1,600 pixels^2^ and 0.55° eccentricity, and thus with a major axis of 50 pixels and minor axis of 41 pixels. For the nontarget ellipse, the orientation was randomly drawn from a von Mises distribution centered at 0 with *κ* = 30 and then divided by 2, approximately equivalent to a Gaussian distribution with mean 0 and a standard deviation of about 5°. The color of the nontarget ellipse was based on a uniformly drawn sample that was used to linearly interpolate between blue and yellow in CIE L*a*b* (CIELAB) color space, with blue as [78 −30 −40], corresponding in RGB space to [0 167 255], and yellow as [78 0 80], corresponding in RGB to [200 130 0]. For each color, lightness was always kept constant at *L* = 78. Indeed, measured luminance was ∼39 cd/m^2^. The target stimulus was specified on a trial-to-trial basis using the Bayesian algorithm described below.

#### Target Stimulus Generation

The orientation and color of the target stimulus were based on the participant’s previous responses according to an adaptive procedure, a type of Bayesian staircase, applied separately for each of the four conditions. We used the Psybayes algorithm (Acerbi, 2016), based on Kontsevich and Tyler (1999), with extentions to include the lapse rate (Prins, 2012). This procedure maintains a posterior distribution over the parameters and updates it after each trial on which the participant pressed one of the two task-relevant buttons. The next stimulus value is chosen to minimize the entropy of the updated posterior given the stimulus, averaged over the participant’s possible responses weighted by their respective probabilities (Kontsevich & Tyler, 1999). Each one of these four Bayesian staircases generated on every trial a unitless value *w* within the range [−0.5, 0.5] that was converted to stimulus values: to $180\frac{w}{\pi }$ degrees in orientation trials and to [*L*, (*w* + 0.5)*a*_yellow_ + (0.5 − *w*)*a*_blue_, (*w* + 0.5)*b*_yellow_ + (0.5 − *w*)*b*_blue_] in color trials. Thus, target stimulus values fell within an orientation range of −30° to 30° and within a blue to yellow range from [78 −30 −40] to [78 0 80]. We defined the space of parameters that Psybayes constructs the posterior on: For *μ*, we used a linear grid of 51 points from −0.5 to 0.5; for *σ*, a logarithmic grid of 25 points from 0.002 to 0.8; and for λ, a linear grid of 25 points from 0 to 0.3.

#### Trial Sequence (Figure 1A)

A trial sequence started with the simultaneous appearance of a feature dimension cue and a spatial cue, presented for 500 ms. The feature dimension cue for orientation consisted of two white line segments, each of length approximately 1 dva, crossing at the center, with orientations tilted ±26.6° with respect to vertical; for color, it consisted of two semicircles (divided vertically, right one yellow, left one blue) joined to form a circle of radius approximately 0.3 dva. Simultaneously, a spatial cue was presented, which consisted of a horizontal line segment of length approximately 0.5 dva emanating from the center of fixation to the left or to the right. We chose 500 ms to ensure sufficient time for the deployment of endogenous feature-based attention (Liu, Stevens, & Carrasco, 2007). Following a delay of 250 ms consisting of the presentation of a central fixation circle of radius 0.12 dva, two ellipses appeared at 2.5 dva to the right and left of a central fixation circle. The stimuli were presented on the screen for 117 ms, followed by another delay period of 250 ms.

After the poststimulus delay, the participant had to respond about the target ellipse via a specific key press out of a total of eight keys (Figure 1A). On any given trial, six of these eight keys are irrelevant. For orientation, the participants were instructed to press one of the two labeled keys for clockwise (CW) or counterclockwise (CCW), using the left keypad for the left spatial cue and the right one for the right spatial cue. For color, they had to press one of two labeled keys to indicate whether the ellipse was more yellow or more blue, also using the left or respectively right keypad depending on the spatial cue. Figure 1B shows all the four possible cue-response mappings. The direction of the spatial cue was randomly drawn on each trial, so participants used their right hand approximately half the time. After the response, auditory feedback was provided for 200 ms: a 1200 Hz tone if the participant had pressed the correct key and a 500 Hz tone if the participant had pressed any of the seven incorrect keys.

#### Training

Before they began the experiment, participants were guided step by step through the different parts of instructions. The experimenter read the instructions on the screen (presented in Mihali et al., 2018, Appendix, Figure A1A) out loud. To remind subjects of the stimulus–response pairings, a sheet with these pairings was posted on the wall of the psychophysics room (Mihali et al., 2018, Appendix, Figure A1B). In total, participants performed 40 training trials: a short orientation-only block (“O”) of 10 trials, a short color only block of 10 trials, and a short switch block (“S”) of 20 trials. The experimenter was present with the participants during the training to observe responses, provide further feedback, and answer questions. Participants repeated the set of all 40 training trials until they achieved a performance greater than 65%.

#### Experiment Structure

After the training, participants performed eight blocks of about 100 trials each in the order O-C-S-S-S-S-C-O or C-O-S-S-S-S-O-C, with 30-s breaks in between blocks. Changes in block type were signaled with a screen with the instruction “In this block, your job is to report ORIENTATION” for O blocks or “In this block, your job is to report COLOR” for C, or “In this block, your job is to report either ORIENTATION or COLOR” for S, with each feature dimension word followed by its associated symbol. In total, participants completed 800 nonaborted trials, approximately 200 in each one of the four conditions, Ori, Col, OriS, and ColS (from S blocks).

### Statistical Analyses

For most metrics, we report median values and 95% bootstrapped confidence intervals. Across 50,000 iterations, we took samples with replacement from and of the same size as the original data with Matlab’s randsample and calculated the median of each of those sets of samples. The the 2.5th and 97.5th quantiles of the distribution of medians across iterations were taken as the 95% confidence intervals.

#### Three-Way Mixed-Design ANOVA

To determine the differences between groups and the two experimental conditions of load and feature, we used three-way mixed-design ANOVA with two repeated measures, since we have one “between-participants” variable (group) and two “within-participants” factors (feature, Ori vs. Col and load, No-switch vs. Switch). Beforehand, we log-transformed the measures that were lower bounded by 0. When we assumed shared parameters between No-switch and Switch and thus we had only one “within-participants” factor, we used two-way mixed-design ANOVA. We implemented the ANOVAs in SPSS with “General linear model: repeated measures.” For post hoc comparisons, we adjust the significance level according to the Sidak correction to $\alpha_{\text{sid}}=1-{\left(1-\alpha \right)}^{\frac{1}{\text{number of comparisons}}}$. For the three-way mixed-design ANOVA, we performed, unless otherwise specified, 12 planned pairwise comparisons in Matlab: Wilcoxon rank-sum tests between groups (one for each condition, four in total) and Wilcoxon signed-rank tests for conditions within a group (four per group, eight in total). We used the Sidak correction for multiple comparisons, decreasing the significance level to *α* = 0.0043 for post hoc comparisons following the three-way mixed-design ANOVA or, respectively, *α* = 0.0127 following the two-way mixed-design ANOVA.

#### Pairwise Correlations

To correct for multiple comparisons when examining the pairwise correlation matrix of the performance measures, we used a method from Nyholt et al. (2004). If *M* is the total number of measures, the number of effective comparisons will be decreased more if the measures are more highly correlated, as captured in a higher variance of the eigenvalues *λ*_obs_ of the correlation matrix, which we calculated with Matlab’s function eig. Then, $M_{\text{eff}}=1+(M-1)\left(1-\frac{\text{var}\left(\lambda_{\text{obs}}\right)}{M}\right)$. As in Nyholt (2004), *M*_eff_ is used in the Sidak correction (a slightly less conservative alternative to the Bonferroni correction), modifying the significance level to $\alpha_{\text{sid}}=1-{\left(1-\alpha \right)}^{\frac{1}{M_{\text{eff}}}}$.

#### Linear Regression

We implemented multivariate linear regression with Matlab’s fitlm.

#### Logistic Regression for Classification

We fit the logistic regression coefficients with Matlab’s glmfit with input “binomial” and the link parameter “logit.” For a given participant, we used the task metrics and the fitted coefficients with glmval to get *p*(Diagnosis), which was then thresholded at 0.5 to predict the 0 or 1 ADHD diagnosis.

#### Stratified 10-Fold Cross-Validation

To assess the use of this logistic regression classifier for out-of-sample prediction, we calculated the cross-validated accuracy. We did stratified 10-fold cross-validation, in which each fold had four participants, two ADHD and two controls; thus, we trained the classifier to find the coefficients over 36 participants and tested over 4 and calculated the mean accuracy across folds. We did 1,000 runs of this stratified 10-fold cross-validation to allow for different random assignments of participants into folds and took the mean accuracy over runs.

### Parameter Fitting

#### Psychometric Curves and Parameters

We fitted psychometric curves to trials on which a participant pressed one of the two relevant buttons; *s* denotes the normalized stimulus value on a given trial (ranging between [−0.5, 0.5]). We use the following form of the psychometric curve (Wichmann & Hill, 2001):

$$p(r=1|s;\mu ,\sigma ,\lambda )=\frac{1}{2}\cdot \lambda +(1-\lambda )\cdot \Phi (s;\mu ,\sigma ),$$ (1)

where *r* = 1 stands for a response “clockwise” (orientation) or for “more yellow” (color). The parameters are the point of subjective equality (PSE or bias), *μ* is the inverse slope (or noise) parameter, *σ*, where both are inputs to the Gaussian cumulative density function (Φ) and the lapse rate (*λ*). We had four conditions, Ori, OriS, Col, and ColS, and thus four psychometric curves.

#### Parameter Estimation and Model Choice

We performed maximum-likelihood estimation of the psychometric curve parameters *μ*, *σ*, and *λ*. The likelihood of a parameter combination is the probability of the data given that parameter combination; we denote the log-likelihood by LL. We assumed that trials are independent of each other, and thus we summed the log-likelihoods across all trials. We fitted orientation and color trials separately; thus the following log-likelihoods apply to either set of trials. In the main model, we assumed that *μ* and *λ* are shared across both load conditions (No-switch and Switch), whereas *σ* might differ. These assumptions had both a practical and a principled motivation. Assuming that parameters are shared between conditions reduced the number of parameters to eight and made parameter estimates more reliable. Moreover, if *μ* reflects an overall bias and *λ* a generic lapsing process, we did not expect them to change with load. For a model without these assumptions, and a model comparison, see Mihali et al. (2018, Appendix, Figures A7 and A8). The log-likelihood for trials in a given feature dimension becomes

$$\begin{array}{l} \text{LL}(\mu ,\lambda ,\sigma_{\text{No-switch}},\sigma_{\text{Switch}})=\log p(\text{data}|\mu ,\lambda ,\sigma_{\text{No-switch}},\sigma_{\text{Switch}})\\ =\underset{\text{No-switch trials }j}{\sum }\text{log}p(r_{j}|s_{j}\;;\mu ,\lambda ,\sigma_{\text{No-switch}})+\underset{\text{Switch trials }j}{\sum }\text{log}p(r_{j}|s_{j}\;;\mu ,\lambda ,\sigma_{\text{Switch}}), \end{array}$$ (2)

where *s*_*j*_ and *r*_*j*_ are the stimulus and the participant’s response on the *j*th trial, respectively. To estimate the parameters, we searched on a grid with 201 values in each dimension: for *μ* linearly spaced from −0.2 to 0.2, for *λ* logarithmically spaced from 0.0001 to 0.3, and for each *σ* logarithmically spaced from 0.002 and 0.6.

#### Reaction Times

For fitting ex-Gaussian distributions to reaction times, we used a custom-made script modeled after an existing software package (Zandbelt, 2014).

#### Data and Code Availability

Clinical data are not available beyond diagnosis labels, experiment code is available upon request, and behavioral data and analysis code are available at https://github.com/lianaan/Perc_Var.

## RESULTS

We attempted to dissociate perceptual from executive deficits in ADHD with a new visuomotor decision-making task with a task-switching component. This task yielded two main measures: TIMO and perceptual variability.

### Task-Irrelevant Motor Output

**Figure 3.**
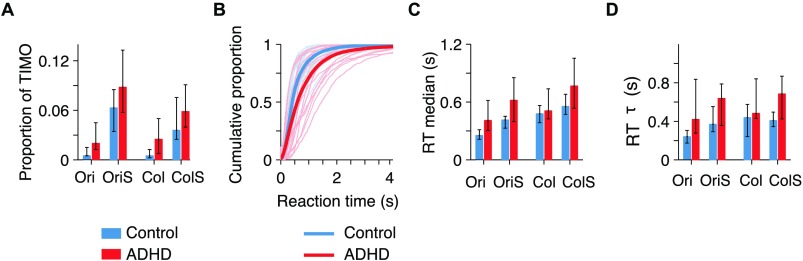
**ADHD participants had higher task-irrelevant motor output and longer and more variable reaction times.** A) Proportion of TIMO across conditions. Here and elsewhere, values represent medians across participants and error bars the bootstrapped 95% confidence intervals. B) Empirical cumulative density functions of reaction times, collapsed across all conditions. Thin lines: individual participants. Thick lines: median for the RT distribution collapsed across all participants in a group. C) Reaction time median by condition and group. Throughout the article, we use RT median because reaction time distributions are not Gaussian. D) Reaction time variability metric, the *τ* parameter from ex-Gaussian distribution fits, by condition and group.

TIMO refers to the trials when participants pressed one of the six irrelevant keys, and hence such responses most likely reflect a failure of proper stimulus-response rule retrieval. TIMO was quite low overall (mean ± sem: 0.06 ± 0.01); ADHD participants produced a higher proportion of TIMO (0.079 ± 0.018) relative to controls (0.041 ± 0.008). Figure 3A presents a breakdown of TIMO by condition. A three-way mixed-design ANOVA on log-TIMO with between-participants variable group and within-participants factors load (No-switch and Switch) and feature (Ori and Col) reveals a significant effect of group, *F*(1, 38) = 8.83, *p* = 0.005, *η*_*p*_^2^ = 0.19, a significant effect of load, *F*(1, 38) = 101.4, *p* < 0.0001, *η*_*p*_^2^ = 0.73, and no significant effect of feature, *F*(1, 38) = 1.62, *p* = 0.21, *η*_*p*_^2^ = 0.04. Neither of the two-way interactions nor the three-way interaction were significant, *p* > 0.06. In particular, the Group × Load interaction was not significant, *F*(1, 38) = 3.72, *p* = 0.06, *η*_*p*_^2^ = 0.09; thus, we did not find that switching between feature dimensions carries a higher cost in ADHD. Next, we performed 12 post hoc planned comparisons: within each group, Wilcoxon signed-rank tests for Ori versus OriS, Col versus ColS, Ori versus Col, and OriS versus ColS, and between groups, Wilcoxon rank-sum tests for Ori, OriS, Col, and ColS. After Sidak correction (*α* = 0.0043), no between-group comparisons were significant, *p* > 0.0046. The within-group load comparisons were all significant, *p* < 0.002. No within-group feature comparisons were significant, *p* > 0.07. Taken together, these results validate TIMO as a metric of interest for executive control.

In the OriS and ColS conditions, the majority of TIMO seemed to be feature errors (Mihali et al., 2018, Appendix, Figure A2B). Relative to the instructions on a given trial, the six irrelevant keys subdivide into two that represent spatial errors, two feature errors, and two both spatial and feature errors. We did not delve into these distinctions since overall TIMO was quite low.

### Reaction Times

ADHD participants showed longer reaction times (RTs; Figure 3B). Three-way mixed-design ANOVA on log RTs revealed a significant effect of group, *F*(1, 38) = 4.72, *p* = 0.036, *η*_*p*_^2^ = 0.11, and significant effects of load, *F*(1, 38) = 84.92, *p* < 0.0001, *η*_*p*_^2^ = 0.69, and feature, *F*(1, 38) = 70.29, *p* < 0.0001, *η*_*p*_^2^ = 0.65. In addition, we found significant Group × Feature, *F*(1, 38) = 4.63, *p* = 0.038, *η*_*p*_^2^ = 0.11, and Load × Feature interactions, *F*(1, 38) = 12.37, *p* = 0.001, *η*_*p*_^2^ = 0.25, but not a significant Group × Load interaction, *F*(1, 38) = 0.08, *p* = 0.77, *η*_*p*_^2^ = 0.002. After Sidak correction (*α* = 0.0043), none of the between-group comparisons were significant, *p* > 0.019. The effects of within-group load and feature on log RTs were all significant both within control and within ADHD, *p* < 0.001. Higher RTs for Col than Ori could be due to the fact that the Ori responses are intuitively mapped to left and right, while the Col responses are arbitrarily mapped as blue to left and yellow to right.

Higher RT variability (or intra-individual variability) in ADHD has been found consistently (Kofler et al., 2013) and has been generally thought to reflect cognitive processes separate from higher median RTs (Castellanos, Sonuga-Barke, Milham, & Tannock, 2006; Kofler et al., 2013, but see Karalunas, Huang-Pollock, & Nigg, 2012, for an opposing account). The term *RT variability* has been used to refer to different aspects of RT distributions (Kofler et al., 2013); here we fitted ex-Gaussian distributions (Leth-Steensen, Elbaz, & Douglas, 2000) and used the *τ* parameter as a measure of RT variability. The *τ* parameter has been shown to capture the tendency of ADHD participants to have a higher proportion of abnormally slow responses (Castellanos et al., 2006; Kofler et al., 2013; Leth-Steensen et al., 2000). Before committing to the ex-Gaussian, we verified that it captures the data better than the log-normal and gamma distributions (see Mihali et al., 2018, Appendix, Figure A4). Three-way mixed- design ANOVA on log *τ* revealed a significant effect of group, *F*(1, 38) = 7.72, *p* = 0.008, *η*_*p*_^2^ = 0.17, an effect of load, *F*(1, 38) = 9.32, *p* = 0.004, *η*_*p*_^2^ = 0.20, and an effect of feature, *F*(1, 38) = 18.85, *p* < 0.001, *η*_*p*_^2^ = 0.33. The only significant interaction was between load and feature, *F*(1, 38) = 14.96, *p* < 0.001, *η*_*p*_^2^ = 0.28. After Sidak correction (*α* = 0.0043), none of the between-group comparisons were significant, *p* > 0.006. Within controls, the effects of load and feature on log RT *τ* were significant for Ori versus OriS and Ori versus Col, *p* < 0.001. Within ADHD, no effects of load or feature were significant, *p* > 0.02. We confirmed the pattern of higher RT variability in ADHD with a nonparametric measure, RT iqr (see Mihali et al., 2018, Appendix, Figure A5).

Overall, we found that ADHD participants had longer and more variable reaction times, consistent with previous work (Douglas, 1999; Kofler et al., 2013; Leth-Steensen et al., 2000). However, RT-related differences across groups are usually difficult to interpret because they might encompass multiple processes, including sensory encoding, decision time, speed–accuracy trade-offs, stimulus–response rule retrieval, response preparation, and response execution (unless some of these processes are disentangled with drift diffusion models; C. Huang-Pollock et al., 2016, Karalunas et al., 2012). The effect of load on RT and RT *τ* does seem to suggest that on Switch trials, more time is spent on executive processes, here stimulus–response rule retrieval, response preparation, and response execution, relative to No-switch trials.

### Psychometric Curve Parameters

**Figure 4.**
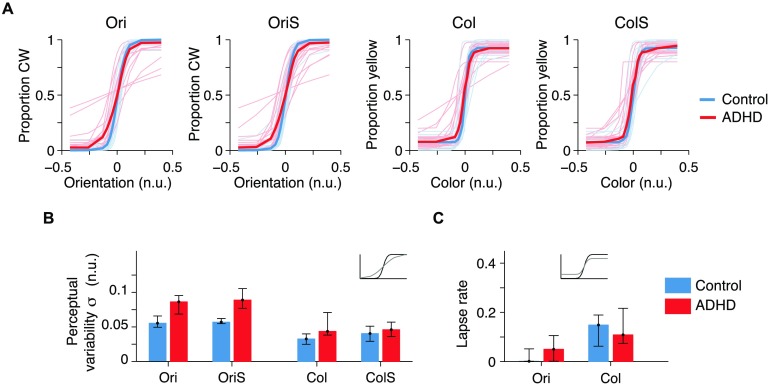
**Fitted psychometric curves and parameters; attention-deficit hyperactivity disorder participants had higher perceptual variability.** A) Psychometric curve fits across all conditions. Here and elsewhere, n.u. stands for normalized units. Thin lines: individual participants. Thick lines: medians for each group. For fits overlaid on top of data, see Mihali et al. (2018, Appendix, Figure A8). B) Perceptual variability parameter values, medians, and bootstrapped 95% confidence intervals. Top inset plot: black psychometric curve has low noise, while the gray has higher noise. C) Lapse rate. Top inset plot: black psychometric curve has low lapse, while the gray has higher lapse.

We confined the following analysis to the trials in which participants pressed one of the two relevant keys. Because of the Bayesian stimulus selection method, each participant received a different set of stimuli for each condition (see Mihali et al., 2018, Appendix, Figure A6), and thus proportion correct is largely stable across conditions and participants (mean ± sem: 0.811 ± 0.007; Mihali et al., 2018, Appendix, Figure A2A) and thus not informative. Instead, we fitted a psychometric curve to non-TIMO trials in each condition (Kingdom, 2009). Thus, the parameters of the psychometric curves captured the differences in performance across conditions and participants. The normalized orientation and color continua spanned the interval [−0.5, 0.5]. Each psychometric curve has three parameters: a point of subjective equality *μ*, perceptual variability *σ*, and a lapse rate *λ* (Figure 4B, 4C; Mihali et al., 2018, Appendix, Figure A8D). Nonzero *μ* represents a tendency to choose one option more than the other. The parameter *σ* is a composite of sensory noise and decision noise and might also reflect the quality of the allocation of spatial attention and of feature attention in switch blocks. Higher *σ* denotes a reduced ability to discriminate between small variations within a feature. The parameter *λ* reflects trials with lapses in attention or erroneous motor output. In our main model, we assumed that *μ* and *λ* are independent of load; we confirmed this assumption by comparing to a model without these assumptions (“full” model) in Mihali et al. (2018, Appendix, Figures A7 and A8). The parameters *σ* and *λ* might trade off against each other, although this is less of a concern in our main model than in the “full” model.

**Figure 5.**
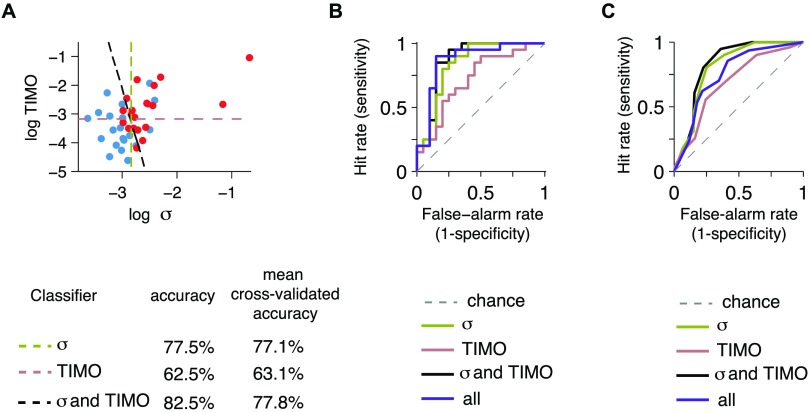
**Logistic regression based on task metrics can classify participants into attention-deficit hyperactivity disorder and controls with accuracies larger than 70%.** A) Dots: combinations of log TIMO and log perceptual variability (*σ*) across participants. Dashed lines: logistic regression classifiers trained on log *σ* only (olive), TIMO only (old rose), and both (black). B) Full receiver operating characteristic curves obtained by varying the diagnosis threshold for the three classifiers in A, as well as for one based on all five behavioral metrics (purple). C) Full ROC curves, this time with stratified 10-fold cross-validation, for the same classifiers as in B.

Three-way mixed-design ANOVA on log *σ* showed a significant effect of group, *F*(1, 38) = 10.56, *p* = 0.002, *η*_*p*_^2^ = 0.22, and a significant effect of feature, *F*(1, 38) = 38.3, *p* < 0.001, *η*_*p*_^2^ = 0.50, but not of load, *F*(1, 38) = 0.97, *p* = 0.33, *η*_*p*_^2^ = 0.025. The effect of Group × Load did not reach significance, *F*(1, 38) = 3.97, *p* = 0.054, *η*_*p*_^2^ = 0.09, and neither did the other two-way interactions, *p* > 0.1, but the effect of Group × Load × Feature was significant, *F*(1, 38) = 6.75, *p* = 0.013, *η*_*p*_^2^ = 0.15. Upon exclusion of the two salient outliers from the ADHD group (Figures 4A and 5A), the three-way mixed-design ANOVA results were highly similar: significant effect of group, *F*(1, 36) = 10.97, *p* = 0.002, *η*_*p*_^2^ = 0.23, and significant effect of feature, *F*(1, 36) = 34.2, *p* < 0.001, *η*_*p*_^2^ = 0.49, but not of load, *F*(1, 36) = 1.79, *p* = 0.18, *η*_*p*_^2^ = 0.05. None of the two-way interactions reached significance, *p* > 0.1, but the effect of Group × Load × Feature was significant, *F*(1, 36) = 6.12, *p* = 0.018, *η*_*p*_^2^ = 0.145. Because the normalization to the (arbitrary) stimulus range is specific to each feature dimension, the values of *σ* cannot be meaningfully compared between orientation and color: A different stimulus range would have changed the *σ* values without changing the observer’s true perceptual variability. Therefore, only the within-feature post hoc comparisons are meaningful, giving a corrected significance level of *α* = 0.0065. Then, the between-group comparisons were significant for both Ori and OriS, *p* < 0.0005, but not for Col, *p* = 0.0083, or ColS, *p* = 0.28. No post hoc comparisons with load were significant either within controls or within ADHD, *p* > 0.01. Higher *σ* for orientation in ADHD could result from worse low-level sensory encoding (e.g., higher neural noise), lower covert endogenous attention, higher decision noise, or even noise in the inference process about the perceptual category. The lapse rate reflects responses that are independent of the stimulus, such as lapses of attention, but could also trade off with the *σ* parameter. Two-way mixed-design ANOVA on log λ showed a large effect of feature, *F*(1, 38) = 28.08, *p* < 0.0001, *η*_*p*_^2^ = 0.43, but no significant effect of group, *F*(1, 38) = 1.72, *p* = 0.19, *η*_*p*_^2^ = 0.04, and no significant Group × Feature interaction. After Sidak correction (*α* = 0.0127), we found that control, *p* < 0.0001, and ADHD, *p* = 0.011, participants tended to lapse more for color than for orientation, possibly because the stimulus–response mapping is less intuitive. Results for *μ* are in Mihali et al. (2018, Appendix, Figure A8B). In conclusion, the parametric variation of low-level stimulus variables combined with stimulus optimization revealed robust perceptual deficits in ADHD, especially for orientation.

A possible cause of the increased perceptual variability in ADHD could be that ADHD participants were slower to learn the task. To check for learning, we fitted two psychometric curves for each condition, one to the first half of the trials and one to the second half. The *σ* parameters across participants, conditions, and time are presented in Mihali et al. (2018, Appendix, Figure A9). Visually, we notice a slight improvement in perceptual variability in the second half of the trials (Mihali et al., 2018, Appendix, Figure A9). To quantify it, we performed a four-way mixed-design ANOVA on log *σ* with time as an additional factor. We found an effect of time, *F*(1, 38) = 12.7, *p* = 0.001, *η*_*p*_^2^ = 0.25. We did not find a significant Group × Time interaction, *F*(1, 38) = 0.42, *p* = 0.52, *η*_*p*_^2^ = 0.01, and thus we have no evidence for a differential learning pattern for ADHD relative to controls.

### Correlations Across Metrics

Next, we asked whether behavioral metrics are correlated with each other (Table 1). For this analysis, we collapsed across groups; per participant, we averaged each behavioral metric across all four conditions. We found that the perceptual variability parameter *σ* is significantly correlated with TIMO, RT median, and RT *τ*, with high effect sizes. Note that the perceptual variability parameter and TIMO were computed from different sets of trials, therefore reducing the probability that their correlation is spurious. In addition, a breakdown of some of these correlations by group, symptom type, and condition is presented in Mihali et al. (2018, Appendix, Breakdown of correlations).

**Table 1.** Pairwise Spearman correlations across log task metrics (both behavioral and clinical)

TIMO	TIMO	RT	RT *τ*	Perceptual variability (*σ*)	Lapse rate (*λ*)	GEC
RT	***ρ* = 0.46**					
	*p* = 0.003					
RT *τ*	***ρ* = 0.42**	***ρ* = 0.84**				
	*p* = 0.007	*p* < 0.0001				
Perceptual variability (*σ*)	***ρ* = 0.41**	***ρ* = 0.55**	***ρ* = 0.57**			
	*p* = 0.0085	*p* = 0.0003	*p* = 0.0002			
Lapse rate (λ)	***ρ* = 0.46**	*ρ* = 0.23	*ρ* = 0.17	*ρ* = 0.28		
	*p* = 0.003	*p* = 0.15	*p* = 0.30	*p* = 0.08		
GEC	***ρ* = 0.53**	*ρ* = 0.25	*ρ* = 0.34	***ρ* = 0.50**	*ρ* = 0.30	
	*p* = 0.0005	*p* = 0.12	*p* = 0.03	*p* = 0.0009	*p* = 0.06	
ACDS	*ρ* = 0.40	*ρ* = 0.31	***ρ* = 0.45**	***ρ* = 0.51**	*ρ* = 0.18	***ρ* = 0.80**
	*p* = 0.01	*p* = 0.05	*p* = 0.004	*p* = 0.0008	*p* = 0.26	*p* < 0.0001

*Note.* Both TIMO and perceptual variability are significantly correlated with several other variables. Boldface denotes significance after multiple-comparisons correction (*α* = 0.0089; see Methods).

So far, we have characterized behavioral differences between ADHD and controls in our task. Next, we asked if behavioral metrics relate to common clinical ones, namely, the General Executive Composite score (GEC), as assessed by the Brief-A questionnaire (self-reported; Roth, Lance, Isquith, Fischer, & Giancola, 2013), as well as the ACDS scores (clinician interview; L. Adler & Cohen, 2004). The GEC and ACDS (Mihali et al., 2018, Appendix, Table A2) are meant to be continuous measures of executive control and symptom severity, respectively. Both GEC and ACDS revealed strong correlations with TIMO, suggesting that TIMO could serve as a behavioral marker of executive deficits. GEC and ACDS were both also strongly correlated with perceptual variability. In addition, ACDS (but not GEC) was correlated with RT *τ*, which provides a graded counterpart of the robust finding of increased RT variability in ADHD (Kofler et al., 2013). A linear regression of GEC as a function of behavioral metrics (*R* ^2^ = 0.38) showed only TIMO as statistically significant (Mihali et al., 2018, Appendix, Table A7A), reinforcing our interpretation of TIMO as reflecting failures of executive function. A linear regression of ACDS as a function of the same metrics (*R* ^2^ = 0.33) only showed significance for RT *τ* (Mihali et al., 2018, Appendix, Table A7B), suggesting that GEC and ACDS, despite being strongly correlated (Mihali et al., 2018, Appendix, Figure A11), could capture distinct aspects of impairment (L. A. Adler et al., 2017). However, the determinant of the correlation matrix of these measures is 0.11, nearing 0 and thus signaling multicollinearity (Dormann et al., 2012). Therefore, we have to be cautious in interpreting the individual contributions of these regressors. Nevertheless, these results suggest that our behavioral metrics capture to some extent the same processes as clinical metrics, while having the advantage of avoiding the potential subjectivity inherent in questionnaires.

### Classification of Participants

Finally, we asked how accurately we can classify a given participant as ADHD or control based purely on behavioral task metrics. Figure 5 depicts these results. A logistic regression using only the perceptual variability parameter yielded a classification accuracy of 77.5%, with a hit rate (sensitivity) of 75% and a false-alarm rate (1 minus specificity) of 20% (Figure 5A). A logistic regression classifier based on TIMO only had an accuracy of 62.5%, with a hit rate of 65% and a false-alarm rate of 40%; using both perceptual variability and TIMO improved the accuracy to 82.5%, with a hit rate of 80% and a false-alarm rate of 15% (Figure 5A). Of note, while these variables are correlated, the determinant of their correlation matrix is 0.82, far enough from 0 that collinearity should not be a problem (Dormann et al., 2012). Adding more regressors (RT, RT *τ*, and lapse) did not yield further improvement (80.0%); this is not surprising in light of multicollinearity. Hence, we consider perceptual variability and TIMO as the main regressors of interest. To assess the use of this logistic classification for out-of-sample prediction, we did stratified 10-fold cross-validation and found mean accuracies of 77.1% with perceptual variability as the only regressor, 63.1% with TIMO only, 77.8% with both perceptual variability and TIMO, and 70.0% with all metrics. The relatively high classification performance suggests that our task has potential as a diagnostic tool.

In addition to thresholding at 0.5 to get diagnosis and, subsequently, accuracy as above, we also thresholded *p*(Diagnosis) at linearly spaced values between 0 and 1 and plotted the resulting receiver operating characteristic (ROC) curves, both without (Figure 5B) and with stratified 10-fold cross-validation (Figure 5C). As expected, the ROC curve for the classifier all metrics shows the best performance (highest area under the curve) without cross-validation, but its performance degrades for out-of-sample predictions in the cross-validated case.

## DISCUSSION

In this study, we dissociated stimulus encoding (perceptual, early) from response selection (executive, late) deficits in ADHD with a new visuomotor decision-making task with a task-switching component. To better separate executive deficits from perceptual and attentional failures, we used eight response keys, six of which were irrelevant on any given trial (TIMO). To assess perceptual precision, we used simple stimuli that varied continuously along one dimension. We used an adaptive stimulus selection method (Acerbi, 2016) that reduced the number of trials needed for accurate parameter estimation (relative to, for instance, the method of constant stimuli); reducing the number of trials is crucial when running the ADHD population. We found differences between ADHD and controls in our main task metrics, TIMO (Figure 3A) and perceptual variability (Figure 4B), as well as median reaction times and reaction time variability (Figure 3C, 3D). We found correlations of these behavioral metrics with clinical metrics (Table 1) and were able to classify participants into ADHD and controls with high (≈77%) accuracy solely on the basis of our main behavioral metrics (Figure 5).

Our finding of higher TIMO in ADHD could be due to more spatial switching errors or more feature switching errors, but it is hard to quantify these contributions since TIMO was overall relatively low. It is conceivable that a less intuitive stimulus–response mapping for orientation distrimination (stimulus oriented toward left/respond with key on the left), or types of stimuli that require spatial integration (Greenberg, Esterman, Wilson, Serences, & Yantis, 2010; Liu, 2003; Mante, Sussillo, Shenoy, & Newsome, 2013; Siegel, Buschman, & Miller, 2015) or cross-modal switching (Haigh et al., 2016), or more complex forms of task switching would produce larger differences on a TIMO-like executive function measure, in line with the executive function impairments widely reported in ADHD (Boonstra et al., 2005; Willcutt et al., 2005).

In line with previous work (Douglas, 1999; King et al., 2007; Kofler et al., 2013; Leth-Steensen et al., 2000), we found that ADHD participants had longer and more variable reaction times. While accuracy was maintained to be approximately stable in all participants, perceptual variability was higher in ADHD, and thus the increased reaction times are not reflective of speed–accuracy trade-offs. In addition, our paradigm allowed us to analyze correlations across individuals between reaction time metrics and other metrics. The correlation between the perceptual variability parameter *σ* and median reaction time is consistent with a drift-diffusion model, in which slower accumulation of evidence simultaneously leads to lower accuracy and longer reaction times. Indeed, many studies have found slower drift rates in ADHD (C. Huang-Pollock et al., 2016; Karalunas & Huang-Pollock, 2013; Karalunas et al., 2012; Lúcio et al., 2016; Ziegler, Pedersen, Mowinckel, & Biele, 2016).

We found higher *σ* in ADHD than in controls. This parameter—which we called the perceptual variability parameter—could be affected both by sensory encoding (affected by attention) and decision processes. Could the differences in *σ* be attributed to either type of process? Sensory and decision noise are usually confounded in the parameters derived from behavior in common discrimination tasks (Gold & Ding, 2013). However, tasks exist in which the influences of sensory and decision noise can potentially be separated (Drugowitsch, Wyart, Devauchelle, & Koechlin, 2016; Keshvari, van den Berg, & Ma, 2012; Lam et al., 2017). Additionally, neural data with high temporal resolution, such as EEG or MEG, could separate perceptual from decision-related variability as early versus late activity relative to stimulus onset (Gonen-Yaacovi et al., 2016; Mostert, Kok, & de Lange, 2015). Decision noise in perceptual decision-making might be related to decision noise on action selection in reinforcement learning models of high-level cognitive tasks. Hauser et al. (2014) found increased decision noise (temperature parameter) in ADHD in a probabilistic reversal learning task and later proposed this to underlie behavioral variability found in ADHD more generally (Hauser, Fiore, Moutoussis, & Dolan, 2016). Our result of increased perceptual variability in ADHD is consistent with this general proposal and extends it to include the possibility of an even lower level correlate of behavioral variability.

Earlier studies examining perceptual function in isolation did not find differences between ADHD and controls (see Fuermaier et al., 2017, for a review). Our result of higher perceptual variability in the ADHD group suggests that the encoding of visual stimuli is less precise than in controls, at least when experimental conditions simultaneously tax other processes. In our case, participants had to allocate either spatial attention or both spatial and feature-based attention as well as employ executive function by maintaining and acting on either two (No-switch) or four (Switch) stimulus–response rules. Earlier studies examining covert spatial attention while attempting to minimize executive load did not find differences between ADHD and controls (Cubillo et al., 2010; C. L. Huang-Pollock & Nigg, 2003; Roberts et al., 2017; Rubia et al., 2010). While perceptual precision and attention might be comparable between ADHD and controls when studied in isolation, it is possible that asking ADHD participants to simultaneously devote brain resources to other processes might allow for differences in perceptual variability to emerge.

### Possible Lower Level Neural Correlates of Behavioral Variability in ADHD

Our results could speak to the question of low-level perceptual components interacting with measured executive control deficits, as we found a significant correlation between the perceptual variability parameter and the executive control metric TIMO. In particular, our results raise the possibility of a shared neural source of perceptual and executive function deficits, such as a lower signal-to-noise ratio in early brain areas. While ideas of lowered signal-to-noise ratio implemented through impaired dopamine and noradrenaline signaling in ADHD have been put forward before, they have been mainly confined to cerebellar, striatal, and prefrontal regions (del Campo, Chamberlain, Sahakian, & Robbins, 2011; Frank, Santamaria, OReilly, & Willcutt, 2006; Hauser et al., 2016). Beyond that, one study found higher neural noise in the visual and auditory cortex of ADHD participants (Gonen-Yaacovi et al., 2016). ADHD participants could have higher perceptual variability in orientation by having less selective orientation tuning of cells in V1; this was the mechanism proposed to underlie decreased orientation discrimination with aging in monkeys (Leventhal, Schmolesky, Wang, & Pu, 2000). The list of regions with lower signal-to-noise ratio in ADHD could include deeper brain structures with roles in selecting relevant sensory stimuli and maintaining stimulus–response rule representations, such as the thalamus (Halassa & Kastner, 2017; Schmitt et al., 2017; Wells, Wimmer, Schmitt, Feng, & Halassa, 2016; Wimmer et al., 2015; Young & Wimmer, 2017), or even lower regions with roles in orienting of attention and behavioral flexibility, such as the superior colliculus (Krauzlis, Lovejoy, & Zénon, 2013; Overton, 2008) or the locus coeruleus (Aston-Jones, Rajkowski, & Cohen, 1999; Devilbiss & Berridge, 2006). However, these regions do not just modulate cortical representations but also receive substantial top-down inputs, so the reduced signal-to-noise ratio could originate from either lower or higher level brain regions.

Based on our data, we cannot establish whether the proposed low-level correlate of behavioral variability is reflective of a diffuse deficit, of frontal-based executive function, or of impairments in endogenous attention reliant on frontoparietal circuits. Nevertheless, our results make the case that low-level perceptual function in ADHD deserves further investigation and that future task designs can easily include assessments of perceptual function—both as behavioral tasks and as questionnaires (Bijlenga et al., 2017; Kim, Chen, & Tannock, 2014; Micoulaud-Franchi et al., 2015; Panagiotidi et al., 2018)—in conjunction with attention and executive function. Using simple rather than high-level cognitive stimuli has the advantage that they can be used in parallel human and animal studies. Studies on animal models of ADHD, such as mouse (Leo & Gainetdinov, 2013; Majdak et al., 2016) and rat (Clements, Devonshire, Reynolds, & Overton, 2014), will provide further insight into the neural circuits implicated in ADHD and how medications can alter these circuits (Hetherington et al., 2017; Mueller, Hong, Shepard, & Moore, 2017).

### Perceptual Variability as a Candidate Diagnosis Marker for ADHD

ADHD diagnosis still relies predominantly on self and sometimes collateral reports, and widely accepted “psychomarkers” (also called “neurocognitive endophenotypes”) and biomarkers are lacking (Thome et al., 2012). For our findings to have implications for clinical practice, it is necessary that our task metrics be predictive of clinical metrics. We found that this was indeed the case. First, based on perceptual variability alone, we were able to classify participants into ADHD and control with cross-validated mean accuracy of 77.0% (including TIMO, 77.7%). Beyond binary classification, we also found strong correlations between behavioral metrics (*σ*, TIMO, and RT *τ*) and clinical ones (GEC and ACDS). Based on these correlations, the behavioral metrics in our task could be considered candidate psychomarkers for ADHD, similar to the performance on the CPT (Ogundele, Ayyash, & Banerjee, 2011), response variability (Castellanos & Tannock, 2002; Henríquez-Henríquez et al., 2015), and drift rate (Salum et al., 2014) and along with potential oculomotor markers like saccade patterns (Munoz, Armstrong, Hampton, & Moore, 2003), microsaccade rate in specific tasks (Dankner, Shalev, Carrasco, & Yuval-Greenberg, 2017; Fried et al., 2014; Panagiotidi, Overton, & Stafford, 2017), pupil size (Wainstein et al., 2017), or eye vergence (Casal et al., 2018). Psychomarkers and oculomotor markers are substantially easier and quicker to test for in large populations relative to other candidate biomarkers, for instance, based on neuroimaging or EEG data (Castellanos & Aoki, 2016; Faraone, Cristian, & Scassellati, 2014; Lenartowicz, Mazaheri, Jensen, & Loo, 2018). While there is a long pipeline from task and metric to clinically useful assay (Hitchcock, Radulescu, Niv, & Sims, 2017; Paulus, Huys, & Maia, 2016), simple behavioral paradigms and modeling applied to ADHD and other disorders could in the long term help refine diagnostic categories and inform and quantify the efficacy of treatment, as is the goal in computational psychiatry more broadly (Montague, Dolan, Friston, & Dayan, 2012; Redish & Gordon, 2016; Wiecki et al., 2015).

## ACKNOWLEDGMENTS

We thank Terry Leon, Michael Silverstein, Saima Milli, and Jonathan Yuh for assistance with protocol preparation, participant recruitment, and clinical data management and analysis. We thank Ili Ma and two anonymous reviewers for comments on earlier versions of this manuscript. In addition, we thank Eero Simoncelli, Marisa Carrasco, Roozbeh Kiani, Mariel Roberts, and members of the Ma lab, especially Maija Honig, Luigi Acerbi, Will Adler, Bas van Opheusden, and Aspen Yoo, for useful conversations. Lenard A. Adler makes the following disclosures for the last three years: Grant/Research: Purdue Pharma, Sunovion Pharmceuticals, Enzymotec, Shire Pharmaceuticals, Lundbeck; Consultant: Sunovion Pharmaceuticals, Shire Pharmaceuticals, Enzymotec, Otsuka Pharmaceuticals, National Football League, Major League Baseball, Rhodes Pharmaceuticals; Shire Pharmaceuticals, Alcobra Pharmaceuticals; Royalty payments (as inventor) from NYU for the license of adult ADHD scales and training materials since 2004. No ownership interests and no speaking fees.

## AUTHOR CONTRIBUTIONS

Andra Mihali: Conceptualization: Equal; Data curation: Lead; Formal analysis: Lead; Investigation: Equal; Methodology: Equal; Software: Lead; Visualization: Lead; Writing – original draft: Lead. Allison Young: Conceptualization: Equal; Data curation: Supporting; Formal analysis: Supporting; Investigation: Equal; Methodology: Equal; Writing – review & editing: Equal. Lenard A. Adler: Conceptualization: Supporting; Funding acquisition: Equal; Methodology: Equal; Project administration: Equal; Resources: Equal; Supervision: Supporting; Writing – review & editing: Equal. Michael M. Halassa: Conceptualization: Equal; Formal analysis: Equal; Funding acquisition: Equal; Methodology: Equal; Supervision: Equal; Visualization: Supporting; Writing – review & editing: Equal. Wei Ji Ma: Conceptualization: Equal; Formal analysis: Lead; Funding acquisition: Equal; Investigation: Equal; Methodology: Lead; Supervision: Lead; Visualization: Equal; Writing – original draft: Lead.

## FUNDING INFORMATION

NYU Langone Medical Center http://dx.doi.org/10.13039/100006962 Award ID: ARSF 53101. Wei Ji Ma, National Institutes of Health http://dx.doi.org/10.13039/100000002 Award ID: R01EY020958.
